# Generalization of Auditory Sensory and Cognitive Learning in Typically Developing Children

**DOI:** 10.1371/journal.pone.0135422

**Published:** 2015-08-12

**Authors:** Cristina F. B. Murphy, David R. Moore, Eliane Schochat

**Affiliations:** 1 Department of Physical Therapy, Speech-Language Pathology and Occupational Therapy, School of Medicine, University of São Paulo, São Paulo, Brazil; 2 Cincinnati Children's Hospital Medical Center and Department of Otolaryngology, University of Cincinnati College of Medicine, Cincinnati, Ohio, United States of America; Birkbeck College, UNITED KINGDOM

## Abstract

Despite the well-established involvement of both sensory (“bottom-up”) and cognitive (“top-down”) processes in literacy, the extent to which auditory or cognitive (memory or attention) learning transfers to phonological and reading skills remains unclear. Most research has demonstrated learning of the trained task or even learning transfer to a closely related task. However, few studies have reported “far-transfer” to a different domain, such as the improvement of phonological and reading skills following auditory or cognitive training. This study assessed the effectiveness of auditory, memory or attention training on far-transfer measures involving phonological and reading skills in typically developing children. Mid-transfer was also assessed through untrained auditory, attention and memory tasks. Sixty 5- to 8-year-old children with normal hearing were quasi-randomly assigned to one of five training groups: attention group (AG), memory group (MG), auditory sensory group (SG), placebo group (PG; drawing, painting), and a control, untrained group (CG). Compliance, mid-transfer and far-transfer measures were evaluated before and after training. All trained groups received 12 x 45-min training sessions over 12 weeks. The CG did not receive any intervention. All trained groups, especially older children, exhibited significant learning of the trained task. On pre- to post-training measures (test-retest), most groups exhibited improvements on most tasks. There was significant mid-transfer for a visual digit span task, with highest span in the MG, relative to other groups. These results show that both sensory and cognitive (memory or attention) training can lead to learning in the trained task and to mid-transfer learning on a task (visual digit span) within the same domain as the trained tasks. However, learning did not transfer to measures of language (reading and phonological awareness), as the PG and CG improved as much as the other trained groups. Further research is required to investigate the effects of various stimuli and lengths of training on the generalization of sensory and cognitive learning to literacy skills.

## Introduction

There is considerable evidence that literacy skills involve both auditory sensory (“bottom-up”) and cognitive (“top-down’) aspects, as demonstrated by the relationship between reading and auditory temporal processing [1–5] and reading and phonological working memory [6–9], respectively. A growing body of research has investigated the effectiveness of auditory and cognitive (working memory and attention) training programs in the rehabilitation of children with phonological and reading impairments [10–17]. Nevertheless, the extent to which auditory or cognitive learning transfers to phonological and reading skills remains unclear [18–21], as do the specific mechanisms that underlie sensory and cognitive learning in children.

Some studies have demonstrated generalization of learning following non-linguistic auditory training to measures of language, including reading, speech perception and phonological awareness [11,13,22]. For instance, Kujala and colleagues [11], using non-linguistic audiovisual training, found that children with dyslexia exhibited not only enhanced electrophysiological mismatch negativity and faster reaction times to sound changes, but also far-transfer to reading skills. Other studies have reported the transfer of musical skills to measures of speech discrimination and phonological awareness [23–25]. For example, Kraus et al [24] evaluated whether “community music programs” lead to improvements in the ability of children to read and process speech sounds. The results demonstrated that, after 2 years, children who were more engaged in the music program developed stronger brain encoding of speech and achieved higher scores in reading tasks. In contrast, both Halliday et al [18] and Murphy et al [19] reported on-task learning, but no generalization to higher-level measures of language skills. In the study of Halliday et al [18], specific auditory training, including tone or phoneme discrimination, was delivered to typically developing groups of children in 12 sessions during a shorter period (4 weeks). In the study of Murphy et al [19], both a typically developing group and a speech-sound disorder group of children were trained on a non-linguistic auditory temporal task in 12 sessions delivered over 12 weeks. The lack of generalization in the Halliday et al. study [18], relative to an earlier study [26] was interpreted as a possible lack of control in the earlier study [26] for the positive influence of interaction with researchers, assistants etc [27]. For that reason, an active control, “Placebo” group was included in the present study. Murphy et al [19], as for a number of studies, reported improved performance on language tasks among all studied groups (including untrained groups), suggesting the operation of a test-retest effect [12, 28], or very rapid perceptual learning [29] rather than generalization of learning per se.

Cognitive training that is focused either on specific skills (e.g. working memory [14–16, 21;30–31], attention [17, 32]), or on broader and mixed skills [12, 33], has been investigated in both typically developing children [29–31] and in children with special needs [12,14,17,20,33] (e.g. attention deficit hyperactivity disorder (ADHD), learning disabilities). Generalization of learning has also been inconsistent in these studies. For example, recent evidence indicates that memory training can lead to significant gains on trained working memory tasks [15–16,21,31]. However, the extent to which working memory learning is transferred to untrained tasks in various domains, such as phonological skills, is not well established [14,16,21,30–31]. Loosli et al [30], for instance, reported significantly enhanced reading performance following working memory training in typically developing children, suggesting shared processes between working memory and reading. On the other hand, while Dunning et al [16] reported improvements in multiple untrained tests of working memory followed adaptive working memory training in children with poor working memory, there was no evidence of improvement in classroom activities involving working memory. For attention training, inconsistent results have again been reported for typically developing children and children with ADHD. Rueda et al [32] investigated the effects of attention training in 4- to 5-year-old children and showed that the trained group exhibited a significant improvement in nonverbal IQ score when compared to the untrained group. This improvement was also reflected in an event-related potential component associated with the ability to resolve conflict. However, a recent meta-analysis concluded that attention training does not significantly improve untrained attention in children with ADHD, which brings the efficacy of intervention programs used for rehabilitation in this disorder into question [21]. No study has investigated the direct effect of attention training on reading skills.

Wright and Zhang [34] proposed that the generalization of learning across tasks and functions should be expected if and only if the tasks depend on the same neural processes. Thus, training that activates or deactivates multimodal association cortices might have broader effects than training that affects only specific sensory areas where generalization across various modalities would not be expected [35]. For example, working memory tasks might activate sensory cortices and the prefrontal cortex [36–37] and thus lead to the transfer of learning from untrained tasks in distinct domains that also depend on the same prefrontal networks, such as reading tasks [38–40]. The characteristics of the trained tasks might also interfere with the transfer and specificity of learning [40]. One example is the involvement of complex cognitive skills in perceptual training tasks. In children, this involvement might act as a limiting process in the generalization of sensory learning because the development of cognitive functions continues until adulthood [41].

Despite these studies involving perceptual (eg. auditory [11,13,22], vision [42–43]) or cognitive (eg. memory [21,30–32], attention [17,32]) learning in children, direct comparison between perceptual and cognitive training on phonological and literacy skills, or even on untrained auditory and cognitive skills, has not previously been attempted. These investigations are important because the greater the number of skills involved in a training task (e.g. perceptual, cognitive and linguistic skills), the greater the difficulty in evaluating which aspects underlie learning generalization. While it may not be possible fully to dissociate different skill domains within a complex training task, in the present research we compared the learning generalization that followed training predominantly aimed at memory, attention or auditory skills in typically developing children. Anticipating the possibility that either or both memory or attention training would have broader effects than would auditory training, we predicted that the learning produced by memory or attention training would transfer more easily to untrained measures relative to auditory learning, which might be more specific to the trained tasks and stimuli. To examine the influence of test-retest and social engagement effects, two additional groups were included: an untrained (‘waiting room’) control group (CG), and a placebo training group (PG) who completed painting and drawing tasks not dependent on time- sensitive responses, but including the same sort of interactions with research staff as the “active” training groups (AG, MG, SG) received. Both far-transfer (reading, phonological awareness) and mid-transfer (untrained memory, sensory and attention tasks) outcome measures were acquired. From a clinical perspective, we expected the results to contribute to a better understanding of the effectiveness of specific training programs on reading disorders, auditory processing disorder (APD), and ADHD. We also expected the results to bear on the specific neural processes that are affected by each type of training.

## Materials and Methods

### Ethics Statement

This study was conducted at the Department of Physical Therapy, Speech-Language Pathology and Occupational Therapy in the School of Medicine at the University of São Paulo and was approved by the Research Ethics Committee of the Analysis of Research Projects of the Hospital das Clínicas, Medical School, University of São Paulo under protocol number 151/13. A written consent form with detailed information about the aim and the protocols of the study was also approved by this ethics committee. All parents provided written informed consent on behalf of their children prior to participation in the study.

### Participants

A total of 60 typically developing children who were enrolled in the first three grades of a private primary school (aged 5 to 8 years) were invited to participate in this study. All children were native Portuguese speakers from the same region of São Paulo city, suggesting a similar socio-economic background. The level of education of the main caregiver was also ascertained. The inclusion criteria were the following: either gender, no familial or personal history of diagnosed or suspected auditory problems, no psychological, otological or neurological disorders or injuries, and no school disciplinary referrals. Additionally, the participants were required to pass an audiometric screen in a quiet room in their school using headphones (pure-tone thresholds < 30 dB HL at 1 kHz, 2 kHz, and 4 kHz). The responses of the parents to questionnaires led to the exclusion of 2 children (neurological disorders), leaving a total of 58 participants.

### Design

The participants were quasi-randomly assigned to one of the following five groups: an attention group (AG), a memory group (MG), an auditory (‘sensory’) group (SG), a placebo group (PG), and a no-intervention control group (CG). The allocation was primarily stratified according to the age of the participants and, secondly, according “reading” and “memory” mean scores. Thus, after the first stratification, we reallocated some participants in order to get approximately equivalent groups in terms of reading and memory skills, but keeping age equivalence as our primary goal. Participant characteristics are shown in Table 1

**Table 1:** Characteristics of the participants.

Variables	AG (n = 11)	MG (n = 13)	SG (n = 12)	PG (n = 13)	CG (n = 9)
Gender (n)					
*Girls (%)*	5	7	6	3	0
*Boys (%)*	6	6	6	10	9
Age (M ± SD)	7 ± 1.2	7.1 ± 0.8	7.3 ± 0.9	7 ± 0.7	7,2 ± 0.4
Caregiver education (yrs)	14.3	15.2	15.7	15.5	14.8
Language tasks (M ± SD)					
*Word reading* Test (%)	40.7 ± 32	66.1 ± 36	63.3 ± 39	49.7 ± 39	81.24 ± 9
Short-term memory					
*Digit Span Test*	3.7 ± 0.7	3.8 ± 0.8	4.3 ± 0.7	3.7 ± 0.9	3.8 ± 0.8
Auditory task					
*Audiological evaluation*	Normal	Normal	Normal	Normal	Normal

AG: Attention group; MG: Memory group; SG: Sensory group; PG: placebo group; CG: Control group; M: median; SD: standard deviation.

There were no significant between-group differences in terms of age (F (4.55) = 0.10, p = 0.98), short-term memory (F (4.54) = 0.97, p = 0.43) or reading (F (4.54) = 2.14, p = 0.09). A difference existed for gender (p = 0.054), given that the groups were not matched according to this characteristic.

A series of tests (‘outcome measures’) were applied before and after the training period to investigate learning transfer to sensory and cognitive skills. Four of the five groups (AG, MG, SG, and PG) received training over 12 weeks. The control group did not receive any form of intervention during the trial interval. However, they still completed the outcome measure tests with a 12 week gap, as did the trained groups. All tests were administered by the same, unblinded experimenter (CFBM).

### Outcome Measures

The outcome measures were categorized as “compliance measures” (i.e., the number of training sessions completed and the progression during training), “mid-transfer measures” (i.e., performance on untrained tasks in the trained domain) and “far-transfer measures” (i.e., performance on untrained tasks in the untrained domain).

#### Compliance measures

The compliance measures included the variables “amount of time (minutes played)”, “total blocks played (blocks of trials)”, and “progression during the game”. These variables were measured throughout the attention, memory and sensory training sessions. The “progression” variable corresponded to the level of difficulty achieved over time for each of the blocks played. The variables “total blocks” and “progression” were measured through the training software (“*Escuta Ativa*”). This software platform included several attention, memory and auditory sensory tasks with similar characteristics in terms of blocks and level of difficulty. It was therefore possible to compare the number of blocks played and progression between training tasks that used similar parameters.

#### Mid-transfer measures

The *visual digit span (forward recall)* [44] task was developed using E-Prime Professional Software. The digit span task began with a sequence of three digits and allowed 12 attempts per series. The children were instructed to verbally repeat the sequence of numbers after viewing the numbers on a computer screen during each attempt. If performance exceeded 50% (i.e. more than six correct attempts within a series), the number of digits in the sequence was gradually increased. The span performance was taken as the length of the last series completed with greater than 50% accuracy.

The *auditory sustained attention* [44] task was developed using E-Prime Professional Software. The duration of the test was approximately 10 minutes, and the test consisted of 398 trials. In each trial, a digit (from 1 to 7) was presented and the participants were instructed to press a button as quickly as possible each time they heard either of the digits 1 or 5 through their headphones. The stimuli were presented diotically at a comfortable listening level that corresponded to a sound pressure level of 70 dB (A). The digit was presented during the first 500 msec of the trial and was followed by an inter-stimulus interval of 1000 msec. Therefore, digits were presented at a rate of 1 digit/1500 msec. The target signal probability was 0.28. Three performance measures were analyzed: correct detection (HIT), false alarms (errors of commission or false detection), and reaction time (RT).

A Brazilian *time-compressed speech test* [45] assessed speech intelligibility using stimuli (words or sentences) compressed as described by Wilson et al. [46]. Briefly, short (≅20 ms) segments of normal-rate speech were deleted digitally, with inter-deletion segments varied in duration to achieve the desired compression ratio. This test has been promoted as a method for assessing temporal acuity in relation to speech intelligibility [47]. In the present study, a list of 50 monosyllabic words with time compression of 70% was applied. The words were presented using a laptop computer (Windows 7 operating system, DirectSound driver) and Trust HS-4100 USB headphones. Performance was analyzed according to the percentage of correctly identified words.

#### Far-transfer measures

The *phonological awareness* test was adapted from a previously reported phonological awareness test [48]. This test includes 6 tasks that occur at the syllable level (syllabic synthesis, syllabic segmentation, syllabic manipulation, syllabic transposition, rhyme and alliteration), and 4 tasks that occur at the phoneme level (phonemic synthesis, phonemic segmentation, phonemic manipulation and phonemic transposition). The performance was analyzed according to the percentage of correct items (five items per task).

The *reading test of single words/adaptation* [49] consists of 30 words that vary according to regularity (regular and irregular words), lexicality (real words and nonsense words), length (short and long stimuli), and familiarity (frequent and non-frequent words). The stimuli were presented with E-Prime Professional software, and participants were asked to read all of the stimuli out loud. Performance was measured according to the percentage of correctly read words. All types of errors were considered as a mistake, such as omissions, substitutions, inversions, word stress, etc.; even refusal to read a specific word.

### Training

The training consisted of twelve 45-minute sessions administered once per week for approximately nine total hours of training. The sessions were performed in groups of approximately 12 children, using laptop computers and headphones, in the multimedia room of the school. The researcher remained with the participants throughout the experiment to evaluate their performance and provide rewards at the end of each session. Details regarding each of the training sessions are provided below. Further details are in the S1 Appendix.

#### Attention training

The attention training included a variety of tasks involving auditory and visual reaction times and sustained, divided and selective attention. The training was performed with headphones connected to a computer at a volume level that was comfortable for the child and delivered using a series of commercial and publicly available Brazilian computerized training games (“*Escuta Ativa*” and “*Pedro no Acampamento*”) and the “*cognitivefun*.*net*” website. For example, in the sustained attention task, the children were instructed to focus and maintain attention on a series of visual stimuli on the computer screen and then select a visual target as soon as it appeared on the screen (“*Pedro no Acampamento*”). The difficulty level varied according to the number of distractors and the time limit available to respond. In the tasks involving auditory and visual reactions (from the “*cognitivefun*.*net*” website), the children were instructed to click on a green dot in the center of the screen as soon as it appeared in the visual test and to respond as soon as they heard a sound in the auditory test. The selective attention task involved a dichotic listening paradigm in which each trial comprised a one-target stimulus followed by two dichotic stimuli (“*Escuta Ativa*”). The children were instructed to choose the right or left symbol on the screen according to the ear in which they heard the target stimulus. The difficulty level varied according to the type of stimulus and the interval between stimuli. The task began with digits, proceeded to words and ended with minimal pair words. Additionally, the interval between the stimuli decreased as the child progressed. The final task was also used to measure the number of blocks played and the progression of each child during the training (compliance measures).

#### Memory training

The memory training focused on phonological, auditory and visuospatial working memory and all tasks involved working memory recall; some of the tasks were both auditory and visual in nature, allowing cross-modal processing. Additionally, the tasks involved various semantic categories and levels of difficulty. The training was performed with headphones connected to a computer at a comfortable volume level for the child and was delivered using two computerized training games (“*Escuta Ativa*” and “*Pedro no Acampamento*”) and the “*cognitivefun*.*net*” website. During the phonological working memory task, the children were instructed to memorize a sequence of auditory digits and then associate that sequence, in reverse order, with the numbers on the screen (“*cognitivefun*.*net*” and “*Pedro no Acampamento*”). The difficulty level varied according to the number of digits (progressing from 3 to 6). During the visual memory task, the children were instructed to memorize a sequence of visual stimuli (shining stones on the screen) and then reproduce that sequence by clicking on the screen directly or indirectly. The difficulty level varied according to the number of stimuli (progressing from 3 to 6) and the interval between stimuli. During the auditory non-verbal memory training task, the children were instructed to memorize a sequence of tones and then reproduce the sequence on a piano on the computer screen (“*Escuta Ativa”)*. The difficulty level varied according to the number of tones (progressing from 3 to 6) and the interval between stimuli. This final task was also used to measure the number of blocks played and the progression of each child during the training (compliance measures).

#### Auditory sensory training

The auditory sensory training focused on the ability to understand speech in noise and on auditory non-verbal skills, frequency discrimination, frequency ordering and backward masking. The training was performed with headphones connected to a laptop computer at a volume level that was comfortable for the child. The training was delivered using the following series of computerized training games: “Escuta Ativa,” “STAR” [50] (backward masking and frequency discrimination), and “Auditory temporal training with non-verbal and verbal extended speech.” The stimuli were presented binaurally at a comfortable intensity using a laptop computer and headphones. A three-interval, three-alternative, forced-choice oddball design was used for both the backward masking and frequency discrimination tasks (“*STAR*”). In the frequency discrimination task, three animated characters were presented visually and sequentially. A 1 kHz pure tone, ‘standard’ stimulus was presented simultaneously with each of two characters, and a higher frequency ‘target’ tone with the remaining character. The objective of the task was to detect the higher frequency by clicking on the corresponding character. During this task, the degree of difficulty was automatically modified by decreasing the difference between the standard and target stimuli through an adaptive staircase assessment. The backward masking task was performed in a similar manner. The standard stimulus was a band-pass noise, centered at 1 kHz and the target was a 20-msec pulse tone presented 50 msec before the same noise. The goal of the task was to recognize the character associated with the pulse tone. The degree of difficulty was modified via adaptive staircase changes in the pulse tone intensity. The frequency ordering task utilized sweep frequencies and was performed using the Auditory Temporal Training with Non-Verbal and Verbal Extended Speech software [13]. This task trained both frequency discrimination and ordering skills. During the task, the participants listened to two or three stimuli (depending on the task phase) and matched the stimuli with the sign on the screen. The task included 18 stages of varying difficulty (i.e., variations in the inter-stimulus interval and stimulus duration). During the speech in noise task, each trial comprised a pair of words in the presence of background, broadband noise. Signal-to-noise ratio varied according to the child’s performance (“*Escuta Ativa*”). The children were instructed to listen to the pair of words and choose whether the words were different or the same. The difficulty level varied inversely with signal-to-noise ratio. This final task was also used to measure the number of blocks played and the progression of each child during the training (compliance measures).

#### Painting training

This placebo training comprised computer-based painting and drawing activities. Free online drawing and paint programs for children were used (“*tuxpaint*.*org*” and “*colorir*.*com*”). During the drawing activities, the researcher selected a specific theme, and the children were instructed to draw something related to that theme using the program tools, such as shapes, colors and special effects. During the painting activities, the children were instructed to paint a specific picture using only primary or secondary colors. The level of difficulty varied according to the theme and the tools available in the program. Given the subjective characteristics of this specific training, no quantitative measure was used to investigate improvements over the course of training (i.e., there were no compliance measures).

#### Control group

this group was the only non-trained group in the present study. While the others groups were trained, they did their normal classroom activities.

### Statistical Analysis

We compared all groups before training, performing One-way ANOVAs for the variables “mean age”, “pre-training task performance” and “minutes played”, and a Chi-Square test for “gender”. The comparison between on-task learning was performed using Student´s t-tests. Two-way mixed ANOVA was used to compare pre and post-training performance as a function of group, with group (AG, MG, SG, PG, CG) as the independent variable and time of testing (pre-test and post-test) as the dependent variable (Group analysis). To identify the differences detected by ANOVA, Tukey’s multiple comparison tests were used. The significance level was set at p < 0.05.

## Results

All trained groups received approximately the same total duration of training (AG = 496 minutes, MG = 523 minutes, SG = 512 minutes, and PG = 519 minutes; 8–9 hours each). There was no significant difference between the groups (F (3,47) = 0.60, p = 0.617).

### Compliance Measures

**Fig 1:**
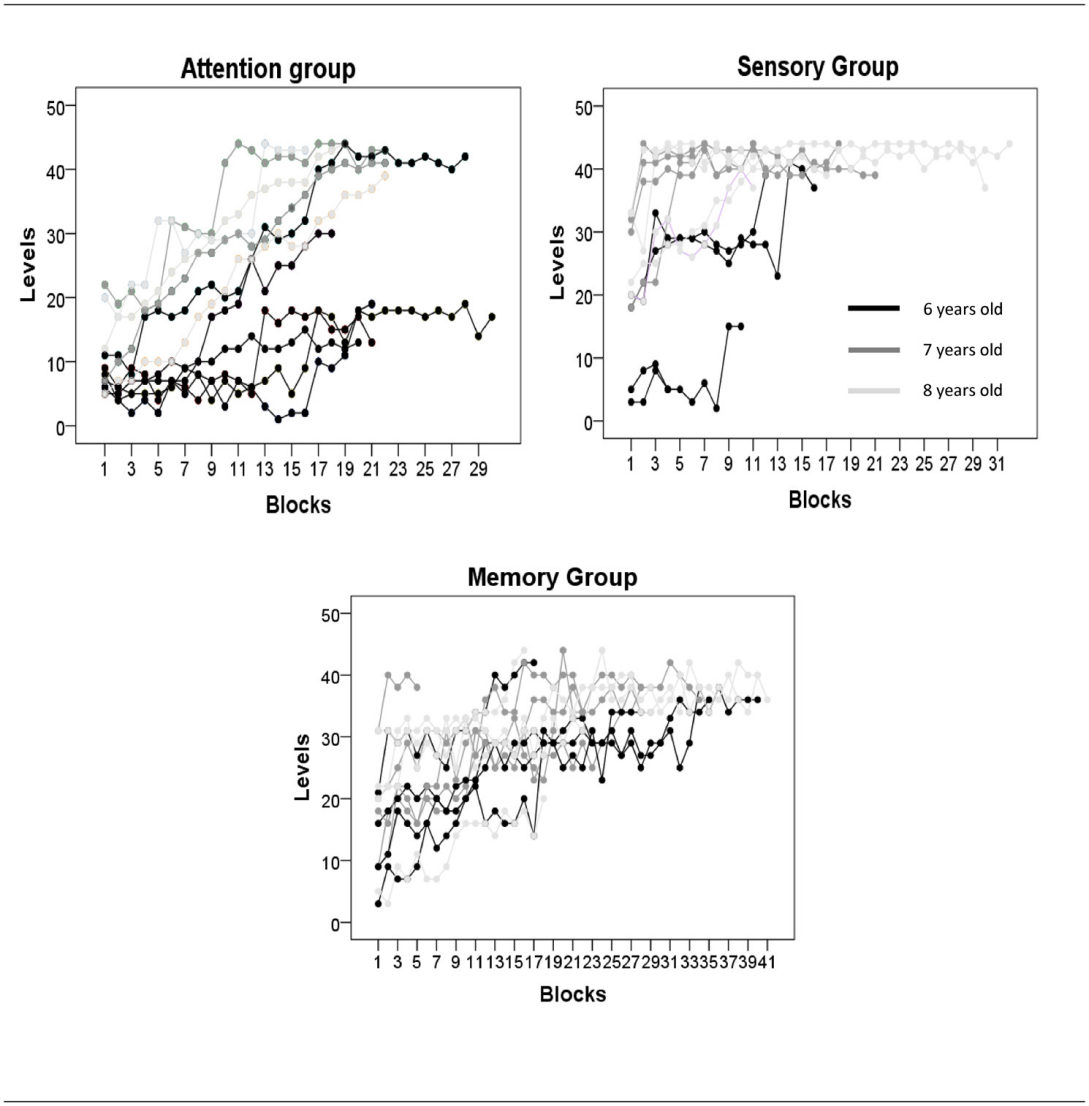
On-task learning. Progression during the training for each child in each group as a function of the computer game ‘level’ achieved and the blocks (number of trials) played. Each line represents an individual participant and different shadings highlight different ages (6,7 and 8 years old).

Fig 1 shows on-task learning during the training phase for 3 of the 4 trained groups (SG, AG and MG), as a function of the (arbitrary game) level achieved and the number of blocks played. The placebo group did not receive quantitative evaluation.

In general, individuals improved with training, and mean performance for each of the 3 trained groups significantly improved with training (all p < 0.001). Children in the Memory and Sensory groups showed converging, improved, performance across successive blocks. For the SG this was clearly due to ceiling performance among those completing more than 14 blocks of training. In contrast, the attention group showed more divergent performance across blocks. Some children improved rapidly, reaching a high performance asymptote in the first half of training, while others improved only very gradually, if at all, across the whole training period. A few children in both groups showed one or more ‘spurts’ of improvement at various points during the first half of training.

Different shadings highlighted that, in general, regardless of type of training, older children seemed to learn faster than younger children. Despite that, in all groups there were some exceptions, demonstrating that even for the same age and the same task, development can occur at different speeds in different children. In the attention task, for instance, two 6 year olds learned rapidly while several of the others seemed to learn slowly. On the other hand, all 7 and 8-year old children improved fast. In the Memory task, one 6 year old seemed to learn rapidly while the two others seemed to learn more slowly, like some of the older children. In the sensory tasks, two 6 year old children were slower while the two others were similar to the older ones.

### Mid- and Far-Transfer Measures

**Fig 2:**
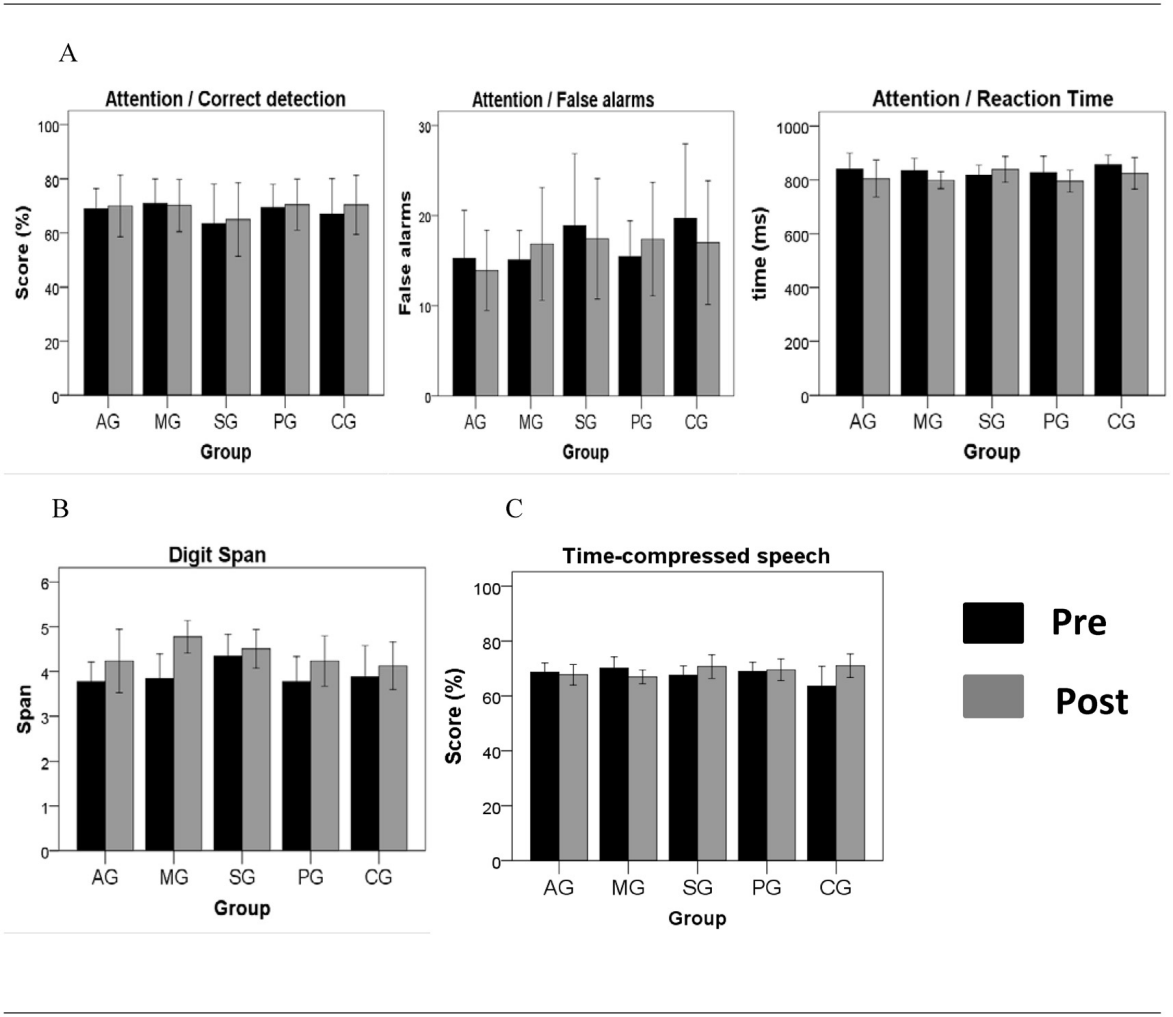
Mid-transfer learning. Pre and post-training mean (± s.e.m.) performance for each of the five groups on measures of (A) auditory sustained attention, (B) digit span and (C) time-compressed speech. Auditory attention performance was measured by correct detection (percentage of the total of number detected correctly), false alarms (total of errors of commission or false detection) and reaction time variables (mean response time for correct detection). Digit span was measured by the last series of numbers completed with greater than 50% accuracy. Time-compressed speech performance was measured by the percentage of correctly repeated words. AG: attention group; MG: memory group; SG: sensory group; PG: placebo group; CG: control group.

Fig 2 shows the mean pre- and post-training performance of the 5 groups in terms of the mid-transfer (auditory attention, memory and speech intelligibility) outcome measures.

For the auditory attention task (Fig 2A), there was no significant difference in the mean proportion of correct detections (p = 0.171) or false alarms (p = 0.958) between the pre- and post-training tests. For the RT, the response time generally and significantly decreased (improved) with training (F(1,51) = 5.62, p = 0.022).

For the digit span test of working memory (Fig 2B), the number of digits recalled increased (improved) from pre- to post-test in all groups, including the CG (F(1,54) = 30.64, p <0.001). There was no main difference between groups (p = 0.537). However, there was a group x retest interaction (F(4,117) = 2.73, p = 0.038). Tukey’s multiple comparison test demonstrated a significantly higher post-training span for the MG (p = 0.02).

For time-compressed speech (Fig 2C), there was no significant overall difference between groups or retest effect.

**Fig 3:**
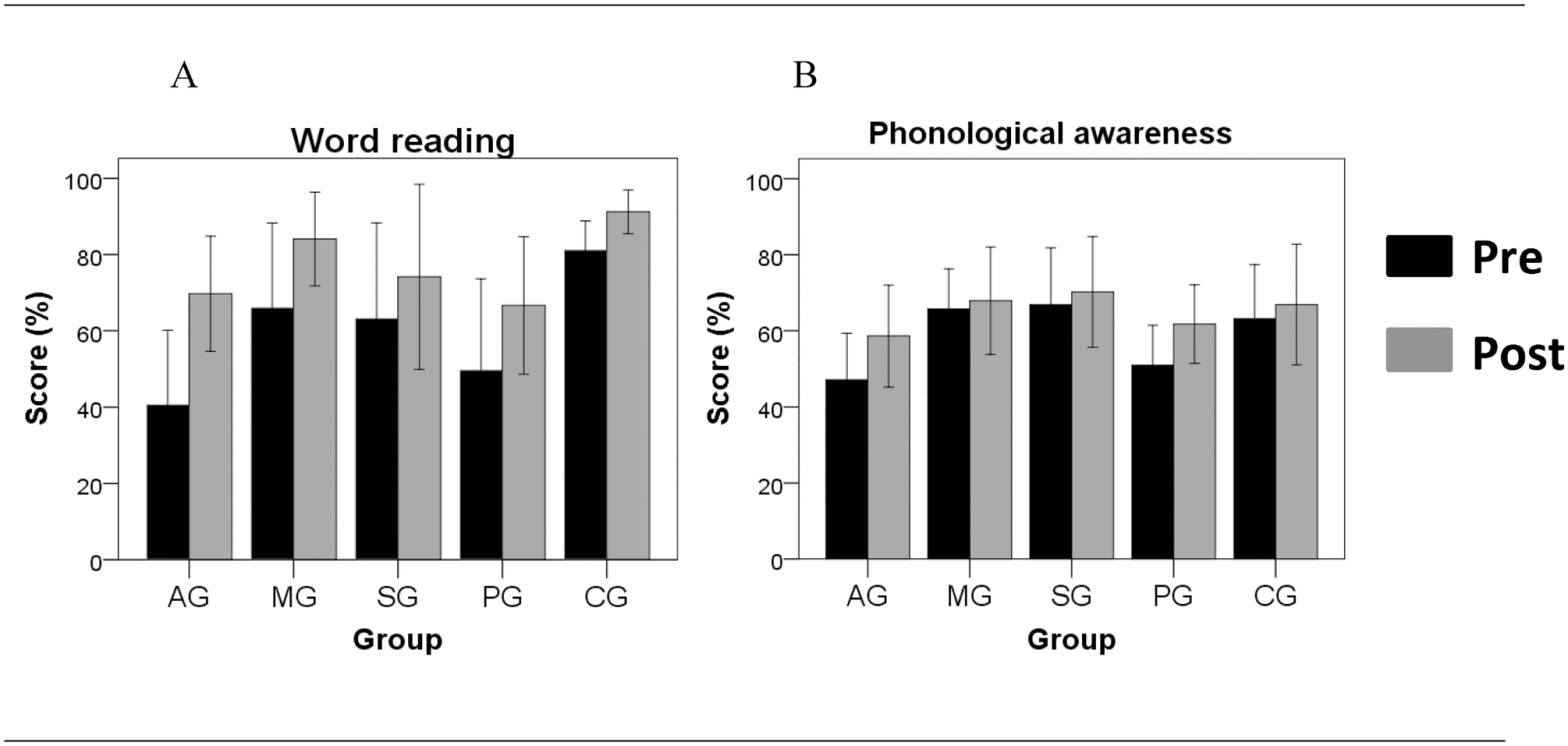
Far-transfer learning. Pre and post-training mean (± s.e.m.) performance for each of the five groups in (A) reading and phonological awareness skills. Word reading performance was measured by the percentage of single words correctly read. Phonological awareness performance was measured by the percentage of correct items in a range of phonological tasks at the syllable and phoneme level. AG: attention group; MG: memory group; SG: sensory group; PG: placebo group; CG: control group.

Fig 3 shows the results from the far-transfer (language) outcome measures.

For the word reading task (Fig 3A), there was a clear retest improvement across all groups (F(1,54) = 57.23, p <0.001). For the phonological awareness task (Fig 3B), there was also a retest effect due to post-training improvements (F(1.117) = 16.12, p<0.001) across groups. No significant interaction between group and retest was observed for either test.

## Discussion

The purpose of this research was to assess the effectiveness of auditory (sensory), memory or attention training on phonological and reading skills, as well as on untrained auditory, memory and attention tasks in typically developing children. The results demonstrated that, although the memory, attention and sensory groups showed significantly marked improvements on the trained tasks, mid-transfer was observed only in the memory group as a modest improvement on an untrained working memory task, relative to the other groups. No far-transfer was clearly established in any group; the PG and CG improved as much as the trained groups on most of the outcome measures.

The improvements in trained tasks observed here corroborated previous research regarding the enhancement of cognitive [21,51] and sensory skills [4, 16] following specific training. However, they also suggested different patterns of learning for each type of task. These different profiles of training may be specific to the tasks used in this study. Since each task was unrelated, the different speeds of training between tasks may simply reflect different task difficulty or age-related effects. Both these factors appeared to be important. Alternately, differences between tasks could suggest that each type of learning is related to distinct training patterns. Further research, controlling for task difficulty and age, is needed to test this hypothesis. In this study, the on-task compliance measures were collected for only one of the trained tasks in each group. Further studies could include two independent compliance measures for each training group to investigate whether this pattern of learning across successive blocks can be attributed to the specific characteristics of the task or to the skill in general.

In addition to the unique patterns of learning found in each on-task training group, learning occurred at different speeds in different children, corroborating previous research [18, 52]. In general, regardless of the type of task, older children learned faster than younger children. Previous research has shown that the performance of children on dichotic listening tests also improves with increasing age [51], indicating that the task is influenced by maturation. However, in each of these cited studies, individual children within an age group had variable levels of performance, so age is not the only, or perhaps even the main source of variability. In the current research, for instance, three 6-year-old children, two in each of the Attention and Sensory groups and one in the Memory group, had better performance than most other children of the same age.

The paradigm of dichotic listening, as used in this study, was also used by Soveri et al [53] to train auditory attention skills in healthy adults. Comparing the effect of different reporting requirements (unforced ‘free recall’; forced-right recall, forced left recall), Soveri et al [53] reported that only forced-left recall improved with training, exhibiting a “modulation” of auditory attention in terms of allocation and transfer of learning to untrained auditory-spatial attention tasks following training. According to the authors, the results supported a hypothesis, elaborated in other studies of Hugdahl and colleagues [54,55], for a higher cognitive engagement in the forced-left condition, produced by competition with the ‘right ear advantage’. Given that attention training in the present study included forced-left recall, but did not find mid-transfer of training, further studies should investigate if modifications of forced-left recall as used by Sovery et al [53] are also effective in training attention in children.

The untrained attention, memory and auditory sensory tasks were applied before and after training to investigate mid-transfer; transfer across tasks under the same trained domain. The results demonstrated that, compared to the other groups, the MG exhibited greater improvement on the Visual digit span task following memory training that included different memory span tasks, suggesting mid-transfer from the memory trained tasks to an untrained memory task. To be precise, improvement on the trained memory tasks that included backward digit span requiring a motor response (pressing the number on a keyboard) was associated with improvement on the outcome memory task involving forward digit span and a verbal response. Other studies performed in typically developing children [22] and in children with ADHD [15] have reported similar results. For example, in a recent study, Karbach et al [31] investigated transfer from a trained memory span task to an untrained memory span task in typically developing children. As in the present research, these authors also reported transfer across the different memory span tasks, suggesting that the learning was not specific to the trained material. This mid-transfer learning likely involved cognitive components that were common to both the trained and the untrained tasks. In a combined working memory and fMRI study, Olesen et al [56] also reported improved performance of untrained digit span following working memory training. The mid-transfer training was associated with increases in task-related prefrontal and parietal activity. The authors suggested that the improvement of functions of a multimodal area could explain how training can affect different cognitive functions, as observed by the improved performance on non-trained memory tasks.

No mid-transfer was observed across the attention tasks or the auditory sensory tasks. Regarding the auditory sensory tasks, the lack of transfer observed in the present study corroborates the results reported by Halliday et al [18]. These authors did not observe transfer across or within auditory or visual stimuli or tasks. They inferred that the lack of learning generalization may have indicated a lack of common procedural (task) learning. This inference might also apply to the present findings, suggesting a more specific effect of sensory training compared to cognitive training, in which mid-transfer was observed. Consistent with this interpretation, vision research has suggested that the extent of brain plasticity following training may be affected by the degree of specificity of the trained tasks [57]. According to this ‘reverse hierarchy’ hypothesis, more specific tasks lead to a more limited transfer, reflecting task processing at more peripheral stages of the nervous system. Conversely, less specific training leads to broader transfer at more central stages. The results of the present study provide some support for this hypothesis by showing lack of transfer for the sensory trained tasks, reflecting a more peripheral level of processing, and limited transfer for the presumably more central memory tasks.

Another, related hypothesis highlights the extent to which the trained task shares sensory components with the outcome tasks. The sensory training in this study focused on auditory temporal processing, involved in speech-in-noise perception, frequency discrimination (at 1 kHz), and backward masking. The auditory outcome task, time-compressed speech, assesses temporal acuity in relation to speech intelligibility. Few studies have investigated the neural correlates of specific auditory training tasks. Other studies on the visual system have suggested that the specificity of perceptual learning to particular trained stimulus attributes may reflect the tuning characteristics of neurons in primary sensory cortices [34]. Supporting that mechanism, a recent auditory study identified a locus of sound frequency selective, synaptic plasticity on an output pathway of auditory cortex, corticostriatal neurons, during the acquisition of auditory frequency discrimination learning in rats [58]. However, despite frequency discrimination being one of the training tasks in the current study, and the overall heterogeneity of the trained auditory tasks, no transfer of training to the compressed speech task was observed. Perhaps the detailed differences between the trained tasks and the outcome measure (e.g. the method of time compression or the resulting speech distortion) were sufficient to prevent mid-transfer of training.

It might be predicted that improvements in Word reading and Phonological awareness tasks would be observed following training, especially cognitive training where a broader transfer would be expected. However, no such improvement was found. There was a near-significant interaction in Word reading between pre-post training and all the trained groups (except the CG), suggesting a possible generalized enhancement. However, rather than a genuine far-transfer of learning, this finding could be due to normal school reading activities, which may have contributed to the improvement of the performance in this specific task. The marked improvement in the PG supported this suggestion. The smaller gain in the CG may have been due to the superior performance of this group compared to the other groups during the initial test. It therefore seems that learning of the trained task, which was observed in all groups, did not transfer specifically to measures of language skills, such as reading and phonological awareness.

Previous studies of phonological and reading improvement following sensory or cognitive training have yielded contradictory results [11,13,14,16,18–22]. Few studies of nonlinguistic auditory training, for instance, have demonstrated improved reading skills [11,13,22]. In addition, the studies were generally not well-controlled, with the inclusion of placebo and non-trained control groups. In this sense, the present results, especially the improvement of the Placebo group followed ‘training’, reinforce the importance of well-designed studies for appropriate conclusions as well as the potential importance of social interaction and general creative activities as possible drivers of learning. In the present results, the influence of maturation on performance of the attention trained task, and the great variability of individual performance may have impacted negatively on the observation of both mid- and far-transfer. The results demonstrate that improvement of memory span, the one process which improved followed memory training, was not sufficient to promote transfer to the related tasks of word reading and phonological awareness.

Although our hypothesis was not confirmed by the measures of far-transfer, the findings suggest that memory learning can mid-transfer to tasks within the same domain. Based on the assumption that learning generalization occurs if and only if tasks depend on the same neural networks [34], a plausible hypothesis is that the memory training and outcome tasks may share neural processes more than the auditory tasks. While the memory training comprised tasks that involved a variety of stimuli, most tasks had the goal of improving memory span, and this characteristic may have led to more focused training. In contrast, the auditory sensory training involved a relatively wide variety of stimuli and tasks, which may have led to a more general auditory training that was not focused on a particular auditory skill. Additionally, as previously mentioned, the auditory outcome task differed from the trained tasks, not only in terms of the stimuli but also in terms of the type of task.

The time course of training is an important parameter to consider in the context of learning transfer [59]. Halliday et al [18], for instance, discussed whether the 6 hr of training in their research was sufficient to observe learning transfer, since previous research reporting successful transfer had delivered training of more than 10 hr [59–60]. Murphy et al [19], delivered non-linguistic auditory training for 9hr and, corroborating Halliday´s findings, found no far-transfer. In the research reported here, the training was also delivered for approximately 9 hr. This may still not be enough to observe learning transfer. In the correction of amblyopia using unilateral eye patching, for instance, Snellen acuity may only return to normal in the patched eye following more than 100 hours of patching [59].

The current study had several other limitations. The sample size of each group was relatively small, which might have reduced the power of the study to reveal more subtle generalization. Furthermore, on-task training measures should ideally investigate performance on all trained tasks throughout the training process to examine whether the pattern of learning for each type of training is specific to the trained task or the trained domain. Further studies should also investigate the effects of various other stimuli and longer lengths of training on sensory and cognitive learning generalization.

## Conclusion

The present study confirmed that attention, memory and auditory training result in learning of the trained task. However, the patterns of learning are not identical for these skills. In terms of the generalization of learning, mid-transfer was observed in the Memory group, suggesting that memory skills are more easily transferred to untrained memory tasks compared with auditory sensory skills. No far-transfer of training to reading or phonological awareness was observed, which questions the efficacy of current cognitive and sensory interventions for remediating language disorders.

## Supporting Information

S1 Appendix Training tasks. (DOCX)
