# Supplementary material for: Generalization of Auditory Sensory and Cognitive Learning in Typically Developing Children
Source: PLoS One. 2015 Aug 12;10(8):e0135422. doi: 10.1371/journal.pone.0135422 (PMC4534328; doi:10.1371/journal.pone.0135422)
Supplement: S1 Appendix — (DOCX) [file pone.0135422.s001.docx]

**TRAINING TASKS**

This appendix provides more information about the trained tasks in each group. The training consisted of twelve 45-minute sessions administered once per week for approximately nine total hours of training. The sessions were performed in groups of approximately 12 children, using laptop computers and headphones, in the multimedia room of the school. The researcher remained with the participants throughout the experiment to evaluate their performance and provide rewards at the end of each session. The greater the number of blocks played, the greater the rewards. Three different trained tasks (involving attention, memory and auditory skills), from the same software (“Escuta Ativa”), were selected to represent the progress of each group along the training (denominated as “compliance measure”). These tasks have similar characteristics in terms of number of levels, blocks and analysis of progress during the training, making comparison between the performances easier.

**Attention training**

The attention training included a variety of tasks involving auditory and visual reaction times and sustained, divided and selective attention. The training was performed with headphones connected to a computer at a volume level that was comfortable for the child and delivered using a series of commercial and publicly available Brazilian computerized training games (“*Escuta Ativa”* and “*Pedro no Acampamento”*) and the “*cognitivefun.net*” website. Each task was performed for approximately 15 minutes in each session.

***Sustained and divided attention task***

In this task, the children were instructed to focus and maintain attention on a series of visual stimuli on the computer screen and then select a visual target as soon as it appeared on the screen (“*Pedro no Acampamento*”). After some levels, children were required to select, simultaneously, two or three targets on the computer screen, dividing attention to different stimuli. The difficulty level varied automatically according to the number of distractors (in order to train divided attention), the time limit available to respond (in order to train reaction time) and the time to focus and maintain attention on some specific stimuli. Children received trial-by-trial feedback on their performance.

***Auditory and visual reaction time tasks***

The visual and auditory reaction time tasks were analogous and both were available through the “*cognitivefun.net*” website. In the tasks involving visual reaction time, the children were instructed to click on a green dot in the center of the screen as soon as it appeared. In the auditory task, children were also instructed to click in the center of the screen as soon as they heard a sound (pure tone). For both tasks, following the response, the reaction time (msec) appeared in the center of the screen as a feedback. Therefore, the children were able to compare their performance in each trial throughout the task and, consequently, reinforce their attention. For false alarm responses, pressing the button prior to the stimulus presentation, children received negative feedback on the screen (“be patient”).

***Selective attention task***

The selective attention task involved a dichotic listening paradigm in which each trial comprised a one-target stimulus followed by two dichotic stimuli (“*Escuta Ativa*”). The children were instructed to choose the right or left symbol on the screen according to the ear in which they heard the target stimuli. The difficulty level varied automatically according to the type of stimulus and the interval between stimuli. The task began with digits, proceeded to words and ended with minimal pair words. Additionally, the interval between the stimuli decreased as the child progressed. The trials were combined in blocks of training. At the end of each block, the software automatically calculated the percentage of right answers and then, lead the player to a higher level (for great percentages) or keep in the same level (low percentages). The task had a total of 45 levels**.** Children received trial-by-trial feedback on their performance. This task was also used to measure the number of blocks played and the progression of each child during the attention training (compliance measures).

**Memory training**

The memory training focused on phonological working memory and auditory and visuospatial short-term memory and all task involved working memory recall. Some of the tasks were both auditory and visual in nature and required cross-modal processing. Additionally, the tasks involved various semantic categories and levels of difficulty. The training was performed with headphones connected to a computer at a comfortable volume level for the child and was delivered using two computerized training games (“*Escuta Ativa”* and “*Pedro no Acampamento”*) and the “*cognitivefun.net*” website.

***Phonological working memory tasks***

Both auditory and visual working memory tasks were performed. In the visual test (“*cognitivefun.net*”), the children were instructed to memorize a sequence of visual digits presented in sequence on the screen, and then to press the numbers, in backward order, using the keyboard. The difficulty level varied according to the number of digits (progressing from 3 to 6). In the auditory test (“Pedro no Acampamento”), the children were instructed to hear the numbers and then reproduce the same sequence with the numbers on the screen. The difficulty level also varied according to the number of digits (progressing from 3 to 6). Children received trial-by-trial feedback on their performance.

**Short-term memory tasks**

During the visual short-term memory task, the children were instructed to memorize a sequence of visual stimuli (shining stones on the screen) and then reproduce that sequence by clicking on the screen directly or indirectly. The difficulty level varied according to the number of stimuli (progressing from 3 to 6) and the interval between stimuli. Feedback was provided for each trial. During the auditory non-verbal memory training task, the children were instructed to memorize a sequence of tones and then reproduce the sequence on a piano on the computer screen (“*Escuta Ativa”).* The difficulty level varied according to the number of tones (progressing from 3 to 6) and the interval between stimuli. This final task was also used as a compliance measure. As for the attention compliance measure, previously described, the trials were also combined in blocks. Therefore, at the end of each block, the software automatically calculated the percentage of right answers, then lead the player to a higher level (for higher percentages) or kept the player at the same level (lower percentages). The task had the total of 45 levels.

**Auditory sensory training**

The auditory sensory training focused on the ability to understand speech in noise and on auditory non-verbal skills; frequency discrimination, frequency ordering and backward masking. The training was performed with headphones connected to a laptop computer at a volume level that was comfortable for the child. The training was delivered using the following series of computerized training games: “Escuta Ativa,” “STAR [47]” (backward masking and frequency discrimination), and “Auditory temporal training with non-verbal and verbal extended speech®.” The stimuli were presented binaurally at a comfortable intensity using a laptop computer and headphones.

***Backward masking task.***

A three-interval, three-alternative, forced-choice oddball design was used for backward masking (“*STAR*”). Three sound-emitting characters were presented, and one character also emitted a 20-msec pulse tone target 50 msec before the noise. The goal of the task was to recognize the character that emitted the pulse tone and the noise. The degree of difficulty was modified via adaptive staircase changes in the pulse tone intensity. There is no time limit in which to respond, and the initiation of each new trial was self-paced.

***Frequency discrimination and ordering tasks***

A three-interval, three-alternative, forced-choice oddball design was used for frequency discrimination tasks (“*STAR*”). Three sound-emitting characters were presented, and one character emitted a sound at a frequency that was different from the other frequencies. The objective of the task was to detect the different frequency by clicking on the corresponding character. During this task, the degree of difficulty was automatically modified by decreasing the difference between the standard stimuli and the target through an adaptive staircase assessment. The initial difference corresponded to a 50% difference in the frequencies of the standard and target tones. There is no time limit in which to respond, and the initiation of each new trial was self-paced.

A frequency ordering task was also performed. The frequency ordering task utilized sweep frequencies and was performed using the Auditory Temporal Training with Non-Verbal and Verbal Extended Speech® software [13]. This task trained both frequency discrimination and ordering skills. During the task, the participants listened to two or three stimuli (depending on the task phase) and matched the stimuli with the sign on the screen. The task included 18 stages of varying difficulty (i.e., variations in the inter-stimulus interval and stimulus duration). There is no time limit in which to respond, and the initiation of each new trial was self-paced.

***Speech in noise task***

During the speech in noise task, each trial comprised a pair of words with the presence of background noise that varied according to the child’s performance (“*Escuta Ativa*”). The children were instructed to listen to the pair of words, binaurally, and choose whether the words were different or the same. The difficulty level varied with signal-to-noise ratio, at 5dB intervals. This final task was also used to measure the number of blocks played and the progression of each child during the training (compliance measures). As the others, at the end of each block, the software automatically calculated the percentage of right answers and then, lead the player to a higher level (for great percentages) or keep the player in the same level (low percentages). The task had the total of 45 levels.

**Placebo training**

The placebo training comprised computer-based painting and drawing activities. Free online drawing and paint programs for children were used (“*tuxpaint.org*” and “*colorir.com*”). During the drawing activities, the researcher selected a specific theme, and the children were instructed to draw something related to that theme using the program tools, such as shapes, colors and special effects. During the painting activities, the children were instructed to paint a specific picture using only primary or secondary colors. The level of difficulty varied according to the theme and the tools available in the program. Given the subjective characteristics of this specific training, no quantitative measure was used to investigate improvements over the course of training (i.e., there were no compliance measures).
